# Bidirectional dynamic threshold SNN for enhanced object detection with rich spike information

**DOI:** 10.3389/fnins.2025.1661916

**Published:** 2025-09-22

**Authors:** Shaoxing Wu, Gang Wang, Yong Song, Yufei Zhao, Ya Zhou, Qingzhu Meng, Yizhao Liao

**Affiliations:** ^1^School of Optics and Photonics, Beijing Institute of Technology, Beijing, China; ^2^Center of Brain Sciences, Beijing Institute of Basic Medical Sciences, Beijing, China; ^3^Beijing Institute of Automation and Control Equipment, Beijing, China

**Keywords:** RGB and event, spiking neural networks, neuromorphic computing, neuron model, object detection

## Abstract

Spiking Neural Networks (SNNs), inspired by neuroscience principles, have gained attention for their energy efficiency. However, directly trained SNNs lag behind Artificial Neural Networks (ANNs) in accuracy for complex tasks like object detection due to the limited information capacity of binary spike feature maps. To address this, we propose BD-SNN, a new directly trained SNN equipped with Bidirectional Dynamic Threshold neurons (BD-LIF). BD-LIF neurons emit +1 and –1 spikes and dynamically adjust their thresholds, enhancing the network's information encoding capacity and activation efficiency. Our BD-SNN incorporates two new all-spike residual blocks, BD-Block1 and BD-Block2, for efficient information extraction and multi-scale feature fusion, respectively. Experiments on the COCO and Gen1 datasets demonstrate that BD-SNN improves accuracy by 3.1% and 2.8% compared to the state-of-the-art EMS-YOLO method, respectively, validating BD-SNN's superior performance across diverse input scenarios. Project will be available at https://github.com/Ganpei576/BD-SNN.

## 1 Introduction

Object detection is a challenging task in the field of computer vision, which aims to determine the location and category of each object in images or videos, forming the foundation for further analysis and processing. In recent years, ANNs have achieved remarkable results in many fields including object detection. However, as model complexity grows, these methods entail significant computational and memory demands, posing challenges for deployment in real-time applications and resource-constrained environments (Howard, 2017). Therefore, there is an urgent need to explore new methods capable of delivering comparable object detection performance to existing ANN methods, while significantly reducing computational costs. A promising approach is to train SNNs directly with surrogate gradient (Srinivasan et al., 2020), which can achieve high performance with few time steps and process both static images and event data efficiently.

With the development of artificial intelligence, SNN, known as the next generation of neural networks (Maass, 1997), has attracted widespread attention due to its unique advantages such as asynchronous discrete event drive, spike activation that is more in line with physiological characteristics, and no floating-point multiplication operations during the operation process. SNNs employ spiking neurons as computational units (Zador, 1997) and convey information through binary spike signals. Information is transmitted via spikes only when the neurons membrane potential, which denotes the internal state variable of the spiking neuron and corresponds to the membrane potential in biological neurons, reaches the excitation threshold (Andrew, 2003). Therefore, during network inference, the floating-point multiplication involved in weight computation and neuron activation in SNNs can be substituted with addition, enabling more efficient and faster computations compared to ANNs (Merolla et al., 2014; Poon and Zhou, 2011). In addition, event-driven computing methods can also show higher energy efficiency on neuromorphic hardware (Davies et al., 2018; Akopyan et al., 2015; Liu et al., 2019). Today, SNN has been widely used in many fields, including object classification (Hu et al., 2024; Zhu et al., 2024; Shan et al., 2024; Wu et al., 2023), detection (Kim et al., 2020b,a; Li et al., 2022), and tracking (Liu et al., 2024; Zhang J. et al., 2023), etc.

However, when dealing with complex regression tasks such as object detection, the accuracy of directly trained SNNs is inferior to that of ANNs (Zhang H. et al., 2023; Hopkins et al., 2018). In object detection tasks, the network must not only identify objects but also precisely delineate their boundaries, necessitating robust feature representation capabilities (He et al., 2019). SNNs encode information via spike emissions, but their inherently discrete signal transmission constrains the networks feature representation capabilities. Compared to the activation feature maps of ANNs, the binary nature of spikes hinders the smooth capture and representation of complex feature variations, impairing the networks ability to discern subtle features and potentially causing information loss (Guo et al., 2024). Recent efforts to directly train deep SNNs for object detection using the surrogate gradient method have achieved higher accuracy than ANN-to-SNN conversion methods, requiring only four time steps compared to hundreds (Su et al., 2023). However, a performance gap persists when compared to ANNs with equivalent network architectures. Some studies have sought to enhance network performance by mitigating information loss in SNNs (Kim et al., 2023; Guo et al., 2023, 2022; He et al., 2024). However, a systematic analysis of this challenge in object detection tasks remains absent.

To address this issue, we employed information entropy theory (Paninski, 2003) into the object detection task and showed that the binary spike activation maps of SNNs carry substantially less information than ANN activations, leading to reduced accuracy. To tackle this challenge, we propose BD-SNN network, designed to comprehensively express the information embedded in input data for efficient object detection. The BD-SNN network integrates two new full-spike residual blocks, BD-Block 1 and BD-Block 2, alongside the new designed bidirectional dynamic threshold neuron model (BD-LIF) employed as the activation unit. Unlike LIF neurons used in traditional SNNs, BD-LIF neurons emit spikes in two distinct modes, –1 and 1, enabling the transmission of diverse information. Additionally, this neuron model dynamically adjusts its spike threshold, with the threshold varying in response to the depolarization rate of the membrane potential (Azouz and Gray, 2000), mirroring the biological principle of inverse proportionality between spike threshold and depolarization rate. Experimental results indicate that the proposed network outperforms those networks through existing ANN-to-SNN conversion methods and direct SNN training methods in terms of accuracy.

In summary, our main contributions are fourfold:

- Through theoretical analysis and experimental validation, we identified the issue of limited information capacity in the activation process of conventional binary spike neurons. The information capacity of the LIF neuron activation map is found to be 3 times lower than that of the ReLU activation map.
- We propose the BD-SNN network, designed to enable SNN-based object detection with enhanced information representation. The network incorporates two new full-spike residual blocks, BD-Block1 and BD-Block2, which are designed to efficiently extract information and fuse multi-scale features respectively.
- We developed a BD-LIF neuron model capable of emitting spikes in two distinct forms, 1 and -1, while dynamically adjusting its threshold. This model is integrated into the BD-SNN network to enhance the information capacity of the spike feature map.
- Experimental results on the COCO and Gen1 datasets demonstrate that BD-SNN, trained with BD-LIF neurons, enhances both information representation and reasoning efficiency. On the COCO dataset, BD-SNN achieves approximately 3.1% higher accuracy than other state-of-the-art methods while requiring only three time steps instead of four.

## 2 Related work

### 2.1 Learning strategies of spiking neural networks

There are two main methods to obtain high-performance deep SNNs. The first method involves converting a pre-trained ANN into an SNN with an identical structure, which is called ANN-SNN conversion (Bu et al., 2023; Rueckauer et al., 2017). However, this method has some inherent defects that are difficult to solve. First, models trained using this method require extended time steps to approximate the accuracy of the activation values in the original ANN (Bu et al., 2023). This prolongs model inference time and escalates energy consumption, counteracting the low-energy design principles of the SNN. Secondly, the limitations of the rate coding scheme (Al-Hamid and Kim, 2020) cause this method to forgo the rich temporal dynamics of the SNN (Deng et al., 2020; Van Rullen and Thorpe, 2001), rendering it unsuitable for dynamic datasets captured by event cameras. These limitations restrict both the practical application and research potential of this method.

The second method is to train SNN using direct training method (Zhou et al., 2024). This method optimizes the network model by backpropagating simultaneously in the time and space dimensions (Wu et al., 2018). The network trained with this method significantly reduces the time steps required for inference (Wu et al., 2019), thereby reducing energy consumption during inference. Furthermore, when processing dynamic datasets, the direct training method's ability to simultaneously optimize both the time and space dimensions allows the trained model to better handle time-varying inputs (Deng et al., 2020). and adapt to diverse real-time application scenarios. These advantages have recently drawn increasing attention to direct training method. In this study, we chose the direct training method to optimize the network model, aiming to fully leverage its advantages in inference efficiency and dynamic data processing.

### 2.2 Energy efficient object detection methods

Currently, mainstream deep learning-based object detection frameworks can be broadly categorized into two types: two-stage frameworks and one-stage frameworks. The two-stage framework is represented by the RCNN series (Girshick et al., 2014). These methods first generate region proposal boxes and subsequently classify and regress them. One-stage frameworks, including the YOLO series (Redmon and Farhadi, 2018), SSD (Wei et al., 2016), and Transformer-based models (Dosovitskiy et al., 2021), adopt a more direct approach by performing object detection and classification within a single network. Although existing ANN-based methods can achieve good target detection results, they all have high energy consumption problems. In practical applications, this high energy consumption limits its application in some scenarios with high energy consumption requirements. Therefore, more and more studies are trying to use SNN to provide an energy-efficient target detection solution with high efficiency and low energy consumption.

The initial approach to object detection using SNN adopted ANN-SNN based conversion method (Kim et al., 2020b,a; Li et al., 2022). This makes it take a long time to infer and is unsuitable for dynamic datasets captured by event cameras. Additionally, there are some hybrid architectures (Johansson, 2021; Lien and Chang, 2022) try to use directly trained SNN backbones and ANN detection heads for object detection, However, these detection heads introduce additional floating point multiplication operations, which destroy the spike-driven nature of SNNs, Moreover, such architectures are incompatible with certain neuromorphic hardware that exclusively supports spike-based operations (Davies et al., 2018; Akopyan et al., 2015; Liu et al., 2019). It was only in the past two years that fully directly trained deep SNN object detection networks have been successfully developed. For instance, EMS-YOLO (Su et al., 2023) represents one of the pioneering successes in this domain. However, it still faces limitations in fully extracting information from data, particularly in complex scenarios, leaving room for further optimization of the models performance.

### 2.3 Information loss in spiking neural networks

In order to improve the performance of SNN in different tasks, extensive research efforts have focused on mitigating information loss in SNNs, leading to the development of numerous methods and strategies. One specific work (Kim et al., 2023) has analyzed the distribution of temporal dynamic information in SNNs during training by estimating the Fisher information of weights, uncovering the impact of temporal information concentration on SNN performance. In InfLoR-SNN (Guo et al., 2023), it is posited that the reset mechanism of SNN membrane potentials overlooks differences between potentials, leading to information loss. To address this, a Soft Reset mechanism and a Membrane Potential Rectifier are proposed to reduce errors. IM-Loss (Guo et al., 2022) suggests that the spike quantization process in SNNs causes information loss and diminished accuracy. To counteract this, it introduces an information maximization loss function designed to optimize information flow within SNNs. MSAT (He et al., 2024) posits that the uniform response of a constant threshold to varying inputs may result in information loss. To mitigate this, it introduces a multi-stage adaptive threshold mechanism to dynamically adjust membrane potential and input thresholds, thereby reducing information loss.

While the aforementioned studies have advanced efforts to mitigate information loss in SNNs, they still quantize membrane potentials into binary spikes. Some works have attempted to leverage non-binary, bidirectional spikes to enhance SNN performance. For example, Spiking-YOLO (Kim et al., 2020b) recognizes that the negative activation regions in leaky-ReLU-based ANN networks occupy a substantial portion of the network, and proposes neurons capable of emitting both positive and negative activations to compensate for information loss during ANN-to-SNN conversion. Similarly, Ternary Spike (Guo et al., 2024) introduces ternary neurons that emit +1 and –1 spikes, carrying richer information. It further proposes trainable spike amplitudes, allowing the network to represent different information during training and convert to standard ternary SNNs during inference. Although these approaches employ bidirectional spiking neurons, they do not provide a theoretical quantitative analysis of the information-carrying capabilities of spiking neurons versus ANN activations, nor do they quantitatively assess the contribution of bidirectional spikes in reducing information loss during inference, particularly for complex tasks such as object detection that require rich information representation.

## 3 Methodology

### 3.1 Information loss in spiking neural networks for object detection

While directly trained SNNs have demonstrated comparable performance to ANNs with reduced power consumption in tasks like object classification, their performance in object detection remains suboptimal when compared to ANNs. We posit that a key limitation lies in the binary spike feature map's insufficient capacity to convey the requisite information for object detection and complex regression tasks, leading to information loss and reduced accuracy. To verify our hypothesis, we employed information entropy theory, integrating it with the membrane potential dynamics at each layer during binary spike neuron inference for theoretical analysis and experimental verification.

The information expression capabilities *R*(*X*) and *R*(*Y*) of discrete random variables *X* and continuous random variables *Y* can be quantified by the information entropy *H*(*X*) and *H*(*Y*) of *X* and *Y*, respectively, which are expressed by the following Equations 1, 2.


(1) $$\begin{array}{l} R\left(X\right)=H\left(X\right)=-\underset{i}{\sum }P\left(x_{i}\right)\log_{b}P\left(x_{i}\right) \end{array}$$

(2) $$\begin{array}{l} R\left(Y\right)=H\left(Y\right)=-\int_{-\infty }^{+\infty }P\left(y\right)\log_{b}P\left(y\right) \end{array}$$

Here, *P*(*x*_*i*_) represents the probability of the random variable *X* taking the value *x*_*i*_, *P*(*y*) denotes the probability density function of *Y*, and *b* is the logarithmic base, typically set to 2, indicating that the information is measured in bits. Building upon the aforementioned formula, we investigated the disparity in information capacity between binary spike neurons and ReLU activation functions in activating feature maps during inference, employing both qualitative analysis and quantitative evaluation.

From a qualitative analysis perspective, the binary spike output is restricted to two states, 0 and 1, which limits its information entropy. Let *FB* denote the binary spike feature map, where *FB*∈*B*^*C*×*H*×*W*^. For each pixel, the output of the spike feature map is binary (0 or 1), and thus the entropy of each pixel, *H*(*FB*), can be expressed as Equation 3.


(3) $$\begin{array}{l} H\left(FB\right)=-P\left(0\right)\log_{2}P\left(0\right)-P\left(1\right)\log_{2}P\left(1\right) \end{array}$$

The output of the spike feature map is limited to two discrete states, hindering its capacity to represent complex information. Consequently, it is unable to effectively represent multi-class data or dynamically changing information, leading to information loss. In contrast, the output of the ReLU activation function is a continuous, non-negative real value, allowing it to convey richer information. Let *FR* represent the ReLU activation feature map, where *FR*∈*B*^*C*×*H*×*W*^. Since each pixel in the membrane potential map can assume any real value, the entropy of the feature map activated by the ReLU function is continuous within the non-negative range, necessitating the use of a probability density function for its definition. The entropy *H*(*FR*) of each pixel can thus be expressed as Equation 4.


(4) $$\begin{array}{l} H\left(FR\right)=-P\left(0\right)\log_{2}P\left(0\right)-\int_{0}^{+\infty }P\left(x\right)\log_{2}P\left(x\right) \end{array}$$

The probability density function *P*(*x*) for positive values must be determined by statistically analyzing their distribution. While the specific information content of the ReLU activation feature map depends on the probability distribution of the membrane potential, it can theoretically represent more information than the binary spike feature map, as it outputs non-negative continuous values.

**Figure 1:**
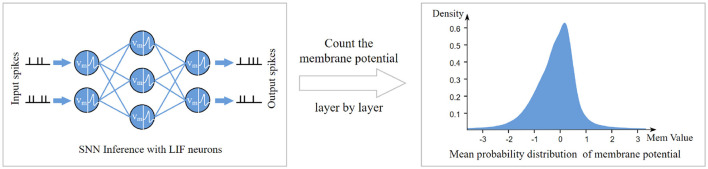
Schematic diagram of the process of obtaining the average membrane potential probability distribution from the network inference process.

When performing quantitative calculations, in order to avoid the sampling error caused by relying solely on the distribution of values from a single layer's membrane potential, we recorded the membrane potential distributions layer by layer during network inference, as illustrated in Figure 1. Subsequently, we divided the membrane potential distributions into three groups, corresponding to the shallow, middle, and deep layers of the network, and averaged them separately to obtain average membrane potential probability distributions for each depth. These averaged distributions were then substituted into Equations 3, 4 to compute the information entropy of the feature maps obtained using LIF neurons and ReLU activation functions. The results are presented in Table 1, indicate that when LIF neurons were used for activation, the average information capacity per pixel was only 0.746 bits, whereas with ReLU activation, it increased to 2.281 bits per pixel. Comparatively, the information capacity of the ReLU-activated feature map was 3.056 times that of the binary spike feature map. This finding underscores the limitations of binary spike feature maps in information representation compared to ReLU-activated feature maps, as quantizing real-valued membrane potentials into binary spikes results in approximately threefold information loss.

**Table 1:** Information entropy of LIF and ReLU activation function in different calculation ranges.

**Calculation range**	***H*(*FB*)**	***H*(*FR*)**	***H*(*FR*)/*H*(*FB*)**
Shallow layers	0.715	2.207	3.087
Middle layers	0.695	2.152	3.096
Deep layers	0.833	2.486	2.984
Global average	**0.746**	**2.281**	**3.056**

*H*(*FB*) denotes the information entropy of LIF neurons, while *H*(*FR*) denotes the information entropy of the ReLU activation function.

Bold values highlight our findings regarding the information entropy under different activation methods.

### 3.2 Design of BD-SNN network

To effectively represent the information in the input features and perform efficient object detection tasks, we propose the BD-SNN network. The BD-SNN network can process both frame-based RGB image data and event image data captured by event cameras for inference, utilizing 1 or –1 spikes to enhance information representation. This feature is mainly based on the design and implementation of the bidirectional variable threshold spike neuron (BD-LIF), the details of which are presented in Section 3.3.

#### 3.2.1 Input representation

**Static image inputs:** Given the spatiotemporal nature of SNNs, when the network input consists of frame-based RGB images, we replicate the image across the time dimension, ensuring that it serves as input at each time step. This method fully leverages the spatiotemporal information processing capabilities of SNNs.

**Event-based inputs:** Event cameras operate fundamentally differently from frame cameras. Each pixel in an event camera can independently respond to changes in light and output these changes in the form of an asynchronous event stream, representing variations in the logarithmic brightness of the pixel. This enables an exceptionally wide dynamic range and superior temporal resolution in the microsecond range. When the logarithmic light level of a pixel exceeds the threshold *V*_*th*_, an event *e*_*n*_ = (*x*_*n*_, *y*_*n*_, *t*_*n*_, *p*_*n*_) is generated. Here, *x*_*n*_ and *y*_*n*_ represent the pixel coordinates, *t*_*n*_ is the timestamp of the event, and the polarity *p*_*n*_ ∈ {−1, 1} represents an increase or decrease in light intensity.

Given a time window ζ, the asynchronous event stream *E* = {*e*_*n*_ ∈ ζ:n = 1, …, N} represents sparse event points in 3D space. In this work, we partition E into segments with a fixed time window dt and map the event points to a 2D representation resembling an image within each segment. The event image generated in each segment is fed into the network as a time step during inference.

#### 3.2.2 Network structure

**Figure 2:**
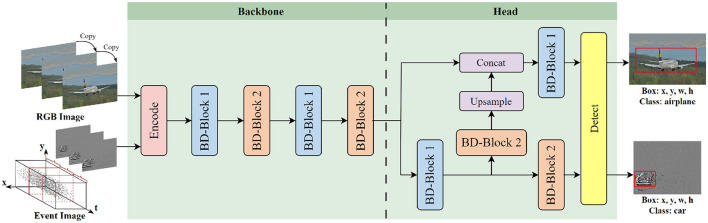
Network structure of BD-SNN. BD-SNN is mainly composed of the backbone and the detection head, which are mainly composed of BD-Block 1 and BD-Block 2.

As shown in Figure 2, BD-SNN consists of two main components: the backbone for feature extraction and the detection head for object detection. Upon input of the RGB or event image into the network, it is first processed by an encoding layer, which includes a convolutional layer and a normalization layer, to convert the input into spikes. Subsequently, a series of BD-Blocks perform information extraction and feature fusion on the resulting spike activation map. Specifically, the BD-LIF neuron learns and integrates weighted inputs from different layers, emitting positive or negative spikes once the threshold is reached, based on the accumulation of membrane potential. We used two different BD-Blocks to extract and fuse features across different dimensions and channels, thereby enhancing the network's robustness. To address the issue of non-differentiable spikes during backpropagation and enable direct training of the network, we employ an alternative gradient (Wu et al., 2018), expressed as Equation 5.


(5) $$\begin{array}{c} \frac{\partial X_{i}^{t,n}}{\partial V_{i}^{t,n}}=\text{sign }|||v_{i}^{t,n}|-v_{th}<a|| \end{array}$$

where *a* is used to limit the range over which the gradient can propagate.

For the object detection task of the SNN model, the main challenge lies in accurately mapping the features extracted from the spike sequence to continuous bounding box coordinate representations. In this study, we feed the final membrane potential of the neuron into the detector to generate anchor boxes of varying scales. After applying Non-Maximum Suppression (NMS), we obtain the category and bounding box coordinates for each object.

**Figure 3:**
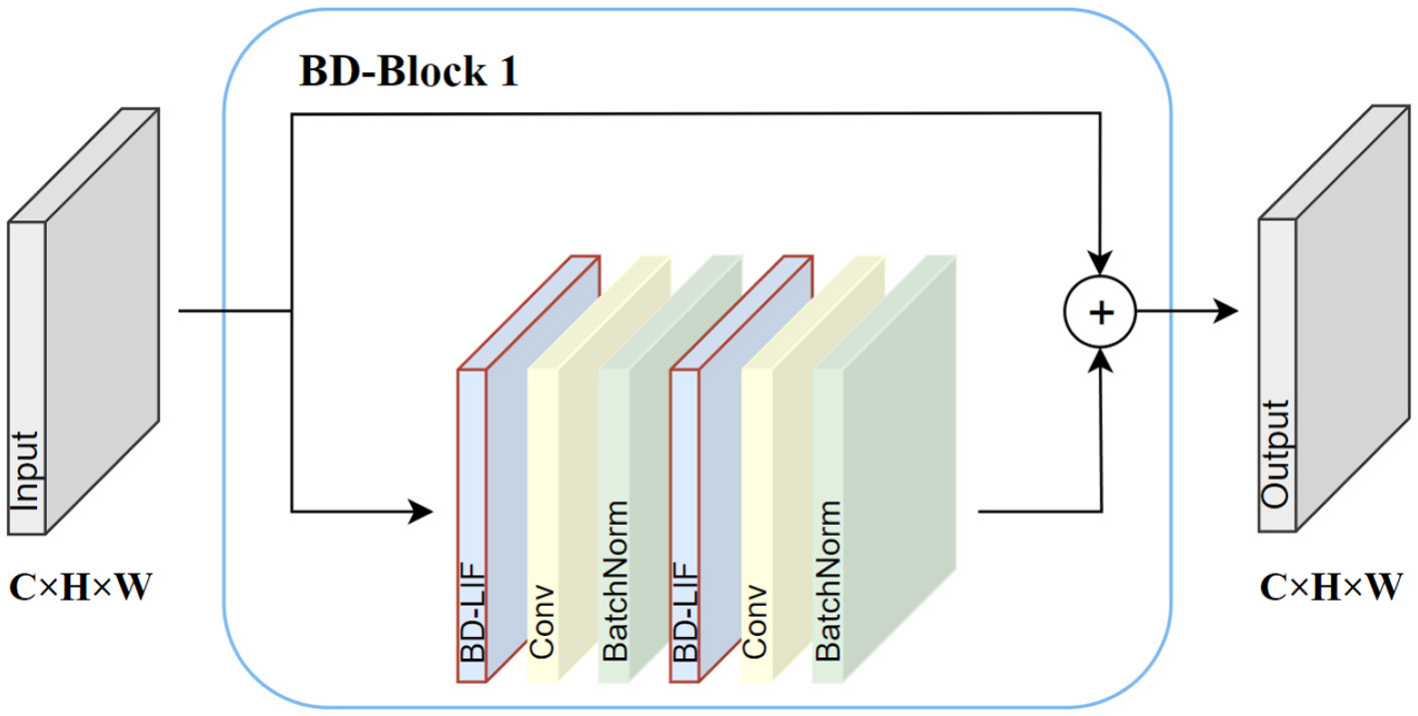
BD-Block 1 internal operation details.

**Figure 4:**
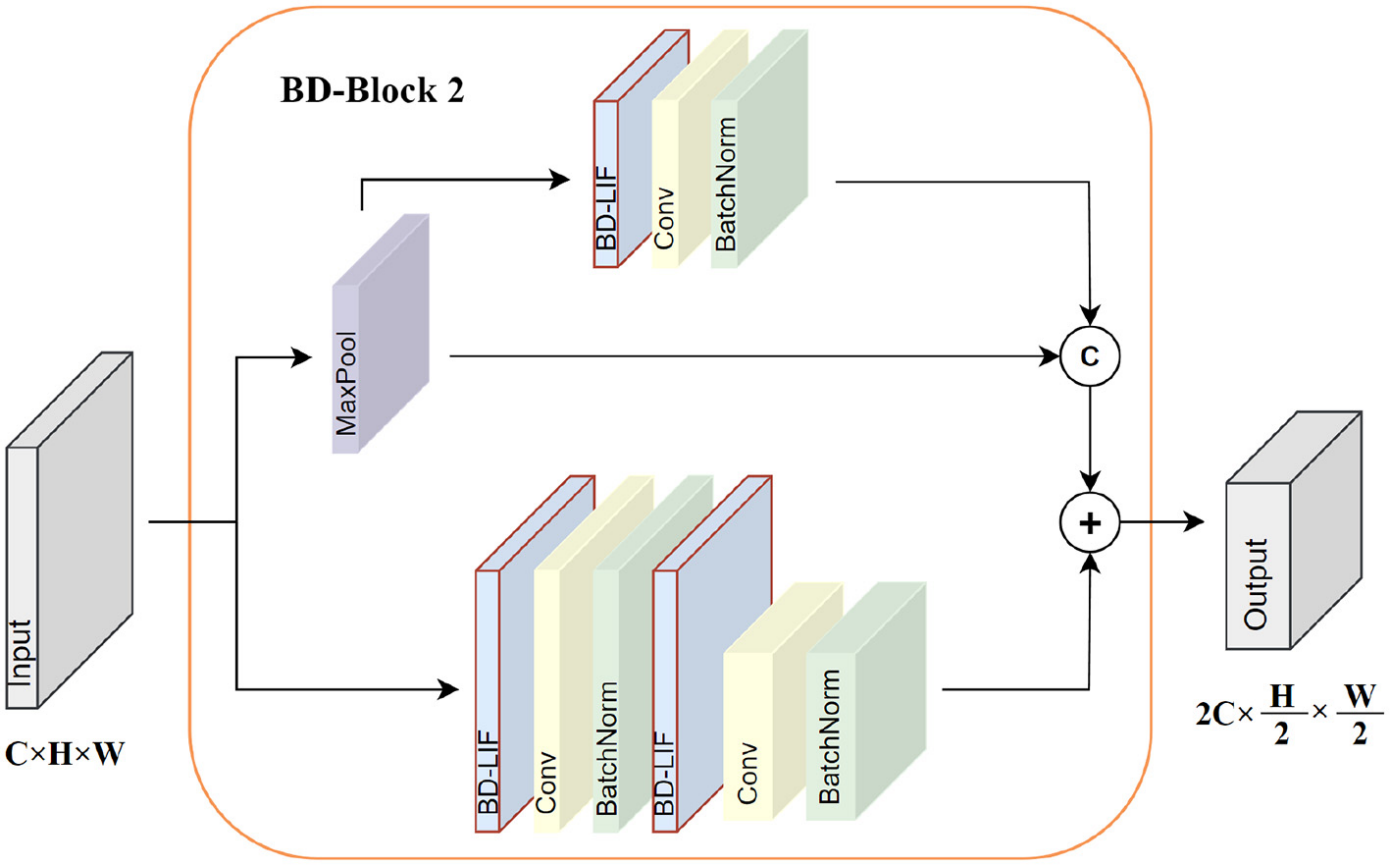
BD-Block 2 internal operation details.

BD-SNN consists primarily of two core modules: BD-Block 1 and BD-Block 2. The operational details of BD-Block 1 are depicted in Figure 3 BD-Block 1 is primarily used to extract features from the spike map, maintaining the input size by performing two consecutive spike activations, convolutions, and normalization operations. Residual connections are also introduced to enhance gradient propagation, enabling more effective training of deeper networks. In addition to the feature extraction function, BD-Block 2 is also responsible for fusing multi-scale features and downsampling the spike map. The specific operation details are shown in Figure 4. BD-Block 2 adopts a CSP-inspired dual-branch structure: one doubles the number of channels in the input spike map and reduces its size by half through convolution operations, while the other branch combines multi-scale features via pooling, convolution, and channel concatenation, resulting in an output with the same shape as the first branch. Finally, the outputs from both branches are combined to produce the final output, integrating multi-scale information.

Unlike EMS-Block (Su et al., 2023), which employs conventional LIF neurons generating unidirectional binary spikes with fixed thresholds, both BD-Block 1 and BD-Block 2 are built upon our proposed BD-LIF neurons. BD-LIF introduces a bidirectional spiking mechanism (outputting −1, +1 instead of 0, 1) and a learnable dynamic threshold adaptation strategy. These neuron-level innovations significantly increase the information capacity of the spike feature maps and alleviate activation inefficiency in deeper layers.

### 3.3 Bidirectional dynamic threshold neuron model

As analyzed in Section 3.1, the conversion of membrane potentials into binary spikes introduces substantial quantization errors, significantly limiting the network model's expressive capacity. To address this issue, we propose a bidirectional dynamic threshold neuron model, termed BD-LIF. Unlike traditional LIF neurons, BD-LIF emits a positive spike (+1) when the membrane potential surpasses the threshold and a negative spike (–1) when it drops below the negative of the threshold. This design improves the information capacity of feature maps while avoiding floating-point multiplications by using subtraction-based operations, preserving the efficiency of spike-driven SNNs.

Furthermore, conventional LIF neurons typically employ a fixed threshold for spike processing. However, the significant variation in inputs across different layers in SNN can constrain the excitation efficiency of neurons under a fixed threshold scheme. In particular, at later time steps of the network, neurons must accumulate a larger number of spikes to surpass the fixed threshold, resulting in information loss and performance degradation. Fortunately, Biological studies have shown that the voltage threshold of biological neurons is not static but dynamically variable. The spike threshold exhibits an inverse relationship with the membrane depolarization rate preceding the spike (Azouz and Gray, 2000), with dynamic threshold changes serving as a critical characteristic of neuronal behavior (Bertrand et al., 2014). The threshold adaptation mechanism of BD-LIF is modeled on this crucial physiological finding. To enable neurons to adapt to the substantial variability in membrane potential distributions across different network layers, we introduced a trainable parameter, α. Which acts as a sensitivity controller. It allows each layer of neurons to learn, during training, how strongly its threshold should respond to changes in membrane potential, effectively tuning the degree of threshold adaptation and improving activation efficiency.

**Figure 5:**
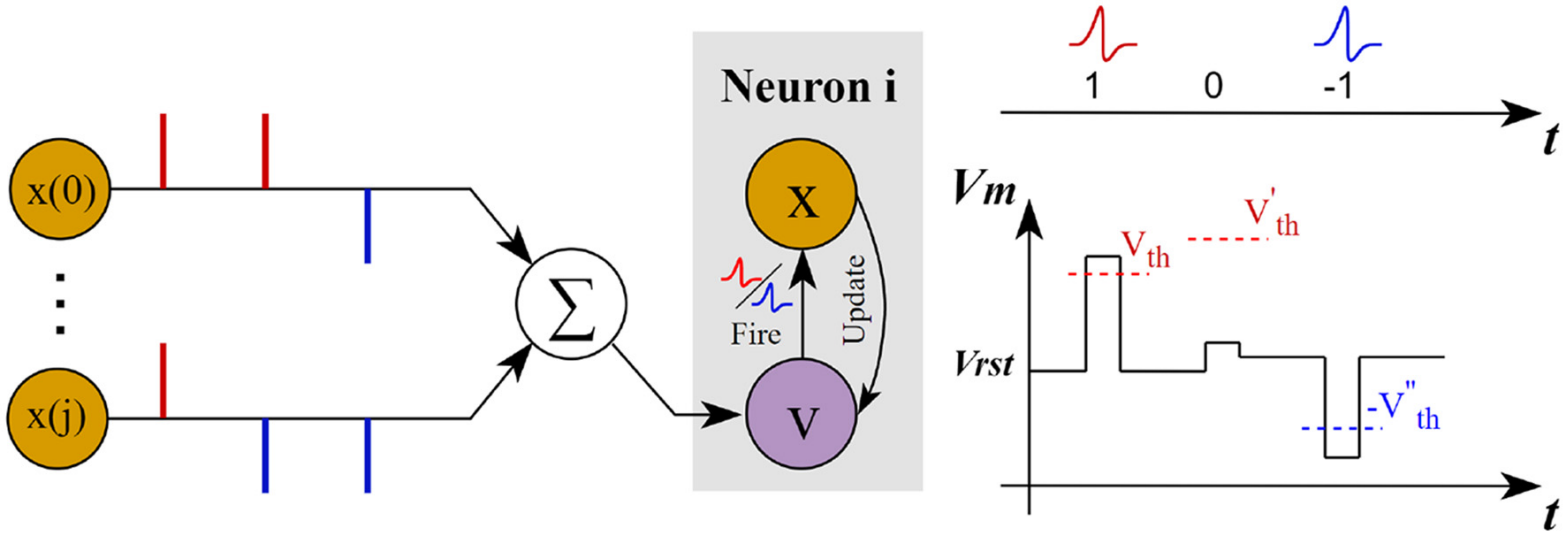
Schematic diagram of the operation of BD-LIF neurons.

As shown in Figure 5, BD-LIF neurons update the membrane potential by integrating both positive and negative spike information across different time steps, while dynamically adjusting their activation threshold. Once the membrane potential surpasses the activation threshold, the neuron will emit either positive or negative spikes accordingly. The detailed neuron model is described by the following Equation 6.


(6) $$\begin{array}{c} V_{i}^{t+1,n+1}=\tau V_{i}^{t,n+1}\left(1-X_{i}^{t,n+1}\right)+\underset{j}{\sum }W_{ij}^{n}X_{j}^{t+1,n} \end{array}$$

In this context, $V_{i}^{\left(t,n+1\right)}$ represents the membrane potential of the i-th neuron in the (*n*+1)-th layer at time step *t*, while τ denotes the integral decay factor. The synaptic input is the sum of the products of $X_{j}^{\left(t+1,n\right)}$ spikes and their corresponding synaptic weights $W_{ij}^{n}$ from the previous layer *n*. The neurons threshold change pattern can be expressed as Equation 7.


(7) $$\begin{array}{c} V_{th}^{\prime }=V_{th}-\alpha \cdot \text{tanh}\left(\psi -b\right) \end{array}$$

In this equation, $V_{\text{th}}^{\prime }$ represents the new membrane voltage threshold derived from the original threshold *V*_th_, while α is a trainable parameter. Additionally, $\psi =\frac{dV_{i}}{dt}$ denotes the depolarization rate of the membrane potential, *b* is a constant that constrains the adjustment range of the membrane voltage to a reasonable interval, and tanh is the hyperbolic tangent function, which maps the depolarization rate of the membrane potential to the range [−1, 1].

Since SNN models a discrete-time process, the calculation method for the neuron depolarization rate ψ can be expressed as follows Equation 8.


(8) $$\begin{array}{c} \psi =\frac{\left|\Delta V_{i}\right|}{\Delta t}=\frac{\left|V_{i}^{t}-V_{i}^{t-1}\right|}{\Delta t} \end{array}$$

Where $V_{i}^{t}$ represents the membrane voltage of the i-th neuron at time step *t*.

Combining Equations 7, 8, the final activation expression of the bidirectional variable threshold neuron (BD-LIF) can be expressed as Equation 9.


(9) $$\begin{array}{c} X_{i}^{t+1,n+1}=\{\begin{array}{ccc} 1, & \text{if} & V_{i}^{t+1,n+1}\geq V_{th}^{\prime }\\ -1, & \text{if} & V_{i}^{t+1,n+1}\leq -V_{th}^{\prime }\\ 0, & \; & \text{otherwise} \end{array} \end{array}$$

**Figure 6:**
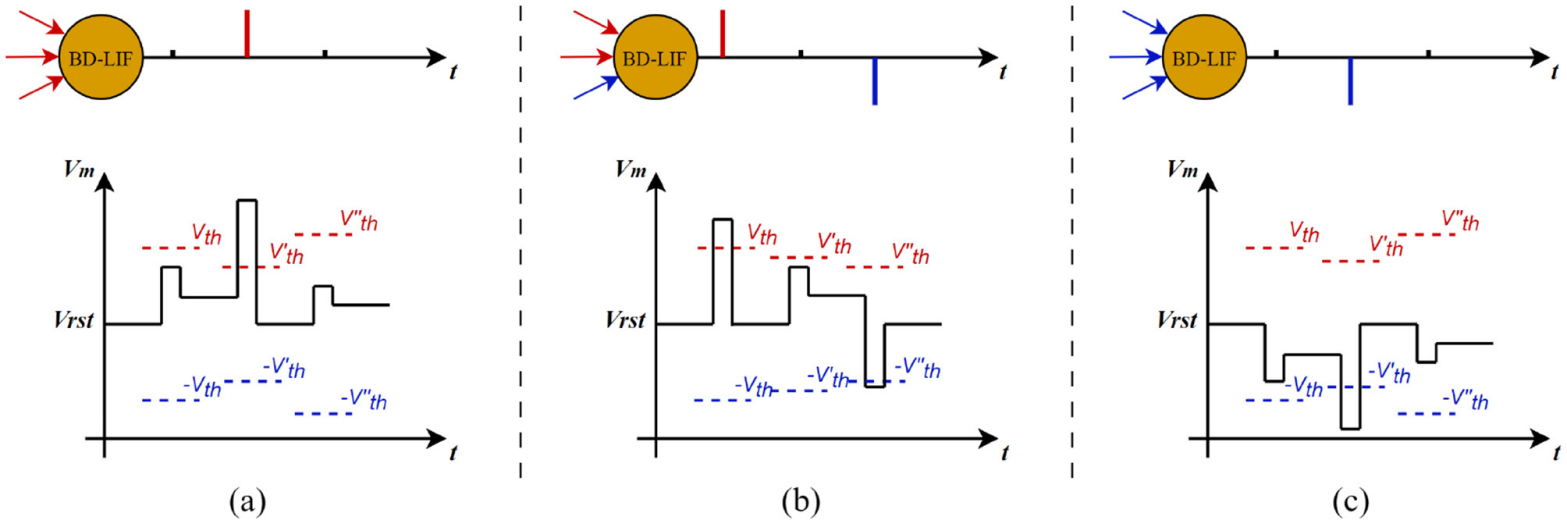
Examples of BD-LIF neuron spike activation. **(a–c)** show how the threshold of the neuron changes with the membrane potential when the neuron only fires positive spikes, fires positive and negative spikes in sequence, and fires only negative spikes, respectively.

As shown in Figure 6, BD-LIF neurons can not only emit both positive and negative spikes but also adaptively adjust their thresholds according to input variations. For instance, in example (a), when the membrane potential increases significantly at the second time step, the neuron's activation threshold decreases accordingly. Conversely, when the membrane potential increase is smaller at the third time step, the activation threshold of the neuron increases.

We hypothesize that BD-LIF neurons enhance the information capacity of spike-activated feature maps. Using the information entropy theory-based method introduced in Section 3.1, we further compare the information capacity of networks employing BD-LIF neurons and conventional LIF neurons across shallow, intermediate, and deep layers. As shown in Table 2, BD-LIF neurons achieve an average information capacity of 1.57 bits/pixel, which is 2.1 times higher than that of traditional LIF neurons. This layer-wise analysis provides quantitative evidence that BD-LIF neurons substantially enhance the feature expressiveness of SNNs during inference.

**Table 2:** Information entropy of LIF and BD-LIF activation function in different calculation ranges.

**Calculation range**	***H*(*FB*)**	***H*(*FL*)**	***H*(*FL*)/*H*(*FB*)**
Shallow layers	0.715	1.524	2.131
Middle layers	0.695	1.519	2.186
Deep layers	0.833	1.657	1.989
Global average	**0.746**	**1.566**	**2.099**

*H*(*FB*) denotes the information entropy of LIF neurons, while *H*(*FL*) denotes the information entropy of the BD-LIF neurons.

Bold values highlight our findings regarding the information entropy under different activation methods.

## 4 Experiment

### 4.1 Implementation details

In order to thoroughly evaluate the effectiveness of the proposed network, we performed experiments on both the static COCO2017 dataset (Lin et al., 2014) and the dynamic Gen1 dataset (De Tournemire et al., 2020) using the BD-SNN network, and compared its performance with several other state-of-the-art methods.

In all of our experiments, we set the number of detection heads to 2 in order to ensure a fair comparison with prior work. For the BD-LIF neurons, we set the reset potential *V*_res_ = 0, the initial threshold *V*_th_ = 0.5 (Su et al., 2023), and the membrane potential decay factor τ = 0.25. The model was trained on two NVIDIA RTX2080Ti GPUs using Python 3.8 and the PyTorch 1.11 deep learning framework, with the SGD optimizer and the cross-entropy loss function. The learning rate was set to 1 × 10^−2^. The network was trained on the COCO2017 dataset for 200 epochs with a batch size of 8, while the model was trained on the Gen1 dataset for 100 epochs with a batch size of 16.

### 4.2 Quantitative evaluation

We conduct a comparative analysis of our network against previous works, evaluating its performance on both the frame-based COCO2017 dataset and the event-based Gen1 dataset.

As shown in Table 3, on the COCO dataset, the previous best method EMS-YOLO, which uses EMS-ResNet34 as the backbone, achieves the highest mAP@0.5 of 0.501 and mAP@0.5:0.95 of 0.301. BD-SNN improves the performance to 0.532 and 0.327 with fewer time steps, representing a effective improvement of 3.1%. As shown in Table 4, on the Gen1 dataset, we trained our model using the same number of time steps as the comparison method, ultimately surpassing it by 2.8%. These experimental results show the advantages of BD-SNN in terms of speed and accuracy.

**Table 3:** Results on the COCO dataset.

**Architecture**	**Model**	**Param(M)**	**Time step**	**mAP@0.5**	**mAP@0.5:0.95**
ANN	Tiny-Yolo	4.3	/	0.258	-
	ResNet18	11.2	/	0.478	0.299
	ResNet34	21.8	/	0.497	0.319
	SSD513 (Wei et al., 2016)	36.0	/	0.504	0.312
ANN-SNNConversion	SNN-VGG16	20.0	64	0.359	0.192
	SNN-InceptionV3	23.9	64	0.413	0.237
	Spiking-Yolo (Kim et al., 2020b)	10.2	3500	-	0.257
	Bayesian Optim (Kim et al., 2020a)	10.2	5000	-	0.259
	Spike Calib (Li et al., 2022)	17.1	512	0.454	0.259
Directly-trainedSNN	SpikeDet (Fan et al., 2025)	22.0	4	0.626	0.462
	SG ResNet (Zhang H. et al., 2023)	22.5	4	0.484	0.296
	EMS-YOLO (Su et al., 2023)	26.8	4	0.501	0.301
	M-SpikeFormer (Yao et al., 2024)	75.0	4	0.503	-
	**BD-SNN** (Ours)	27.0	**3**	**0.532**	**0.327**

“/” denotes that the attribute is not applicable to the corresponding method, while “-” indicates that the value is not reported in the reference.

Bold values emphasize the results obtained by our proposed method.

**Table 4:** Results on the Gen1 dataset.

**Architecture**	**Model**	**Param(M)**	**Time step**	**mAP@0.5**	**mAP@0.5:0.95**
ANN	YOLOv3-tiny (Redmon, 2018)	10.2	/	0.312	-
	Asynet (Messikommer et al., 2020)	11.4	/	-	0.145
	Aegnn (Schaefer et al., 2022)	20.0	/	-	0.163
	Inception+SSD (Iacono et al., 2018)	-	/	-	0.301
	MatrixLSTM (Cannici et al., 2020)	61.5	/	-	0.310
SNN	SpikeDet (Fan et al., 2025)	22.0	5	0.692	0.465
	MobileNet-64+SSD	24.3	5	-	0.147
	VGG-11+SSD	12.6	5	-	0.174
	DenseNet121-24+SSD (Cordone et al., 2022)	8.2	5	-	0.189
	Spiking-Yolo (Kim et al., 2020b)	7.9	500	0.442	-
	Tr-Spiking-Yolo (Yuan et al., 2024)	7.9	5	0.453	-
	EMS-YOLO (Su et al., 2023)	14.4	5	0.541	0.311
	SFOD (Fan et al., 2024)	11.9	5	-	0.321
	**BD-SNN**(Ours)	14.5	5	**0.569**	**0.326**

“/” denotes that the attribute is not applicable to the corresponding method, while “-” indicates that the value is not reported in the reference.

Bold values emphasize the results obtained by our proposed method.

### 4.3 Ablation studies

We performed ablation experiments to assess the impact of the proposed BD-LIF components on both static and dynamic datasets. Specifically, we conducted experiments on the COCO and Gen1 datasets to evaluate the effects of BD-SNN using traditional LIF neurons, neurons with bidirectional spike features only, neurons with dynamic threshold features only, and neurons incorporating both features simultaneously. The experimental results are presented in Table 5. The results show that each component of the BD-LIF neuron model contributes to the enhancement of network performance. This further verifies that the SNN network using the proposed BD-LIF neurons is more competitive when processing traditional frame image data and event data.

**Table 5:** Ablation study of BD-LIF on each characteristic.

**Dataset**	**Bidirectional spike**	**Dynamic threshold**	**Results**	
			**mAP@0.5**	**mAP@0.5:0.95**
COCO			0.498	0.302
	✓		0.525	0.323
		✓	0.506	0.303
	✓	✓	0.532	0.327
Gen1			0.537	0.310
	✓		0.560	0.321
		✓	0.548	0.315
	✓	✓	0.569	0.326

### 4.4 Qualitative analysis

**Figure 7:**
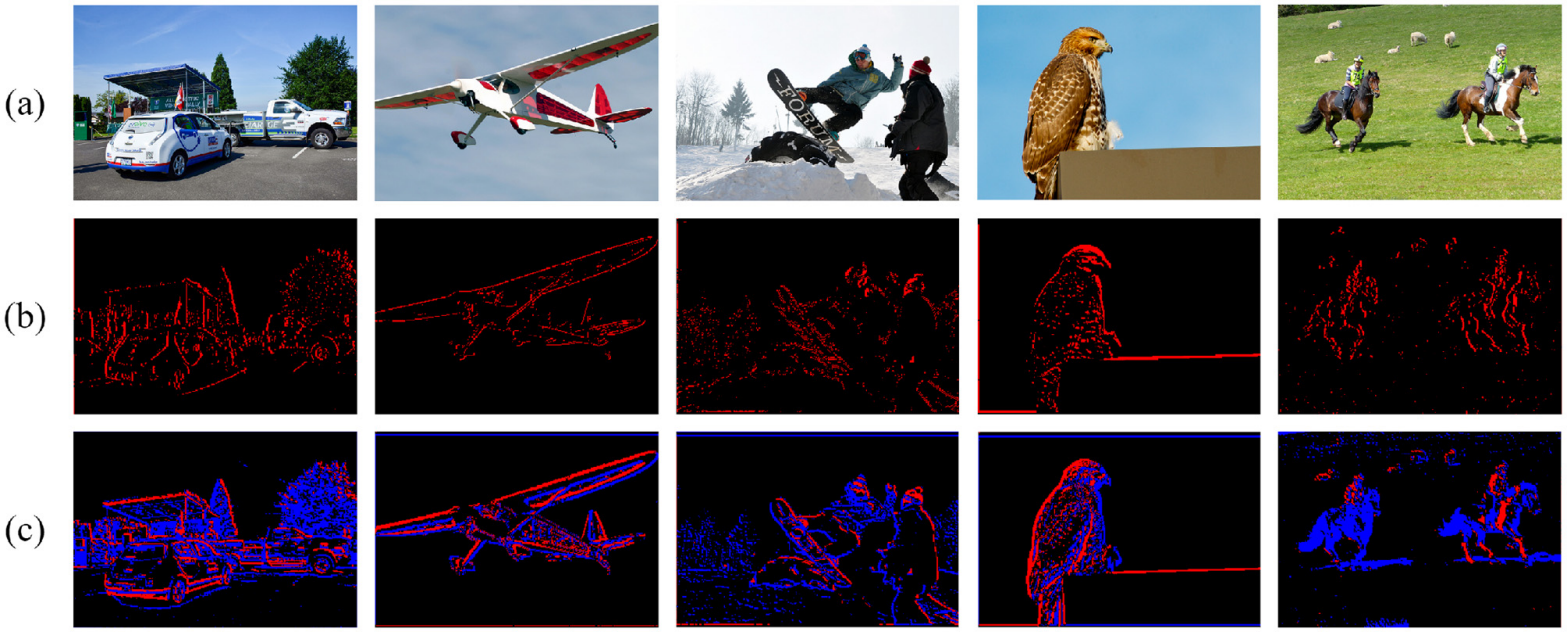
Spike activation diagrams when the network uses different neurons. **(a)** Is the original image, **(b)** is the spike activation diagram when using LIF neurons, and **(c)** is the spike activation diagram when using BD-LIF neurons. Images are from the COCO 2017 dataset (Lin et al., 2014).

To intuitively illustrate that BD-LIF enhances the information-carrying capacity compared to traditional LIF neurons during the network inference process, we analyzed the inference results of networks utilizing different types of neurons on static datasets. Specifically, we extracted several activation maps from the shallow layers of the BD-SNN network and visualized these feature maps, as shown in Figure 7. It can be seen that, compared to traditional LIF neurons, BD-LIF neurons can flexibly utilize two distinct spike modes to represent key image details with greater precision. For instance, in the third column, the network using LIF neurons failed to accurately identify the body contours of the two individuals in the image, leading to the loss of critical details. The network employing BD-LIF neurons not only clearly delineated the body contours of the two individuals through two distinct spike forms, but also precisely captured the ski boundaries. These results demonstrate that BD-LIF neurons enhance the network's sensitivity to fine details and complex features, thereby facilitating superior performance in object detection tasks.

## 5 Conclusion

In this paper, we propose a Bidirectional Dynamic threshold SNN named by BD-SNN, which is capable of emitting both 1 and –1 spikes to convey richer information during inference. This addresses the challenge of limited feature information extraction from neuronal membrane potentials in traditional SNN-based object detection networks, thereby effectively enhancing detection accuracy. Specifically, we designed two new all-spike residual blocks, termed BD-Block, to efficiently extract information and fuse features, and incorporated a new type of spiking neuron, BD-LIF, within these blocks. This neuron can emit both 1 and –1 spikes to enhance the information capacity of the spike activation feature map, while also adaptively adjusting its threshold in a biologically plausible manner to optimize activation efficiency. Experimental results show that BD-SNN improves the accuracy of state-of-the-art method (EMS-YOLO) by 3.1% and 2.8% on the COCO2017 and Gen1 datasets, respectively. We believe that this work provides new insights into enhancing SNN performance in object detection and further expands the potential of spiking neurons for spatiotemporal information processing.

## Data Availability

The original contributions presented in the study are included in the article/supplementary material, further inquiries can be directed to the corresponding authors.
